# Drug-tolerant persister cells in lymphoid malignancies: from mechanisms to therapeutic opportunities

**DOI:** 10.3389/fonc.2026.1713277

**Published:** 2026-07-15

**Authors:** Meng Li, Suping Tang, Peng Yang, Hao Zhou

**Affiliations:** 1 Institute of Hematology, Union Hospital, Tongji Medical College, Huazhong University of Science and Technology, Wuhan, China; 2 Department of Breast and Thyroid Surgery, Union Hospital, Tongji Medical College, Huazhong University of Science and Technology, Wuhan, China

**Keywords:** cancer stem cell, chemotherapy resistance, drug-tolerant expanded persister, drug-tolerant persister, lymphoid malignancies

## Abstract

Therapy resistance and relapse remain major obstacles in the treatment of lymphoid malignancies. While the cancer stem cell (CSC) hypothesis has long served as a conceptual framework for understanding chemoresistance, evidence for its direct applicability in lymphoid malignancies is still limited. More recently, drug-tolerant persister (DTP) cells have emerged as an important model for exploring resistance mechanisms, offering complementary perspectives beyond the CSC paradigm. In this review, we summarize recent advances in the study of DTP cells in lymphoid malignancies, including their progression to drug-tolerant expanded persister (DTEP) cells. We discuss experimental models and methodological approaches for investigating DTP cells, as well as the underlying mechanisms of persistence and resistance, which encompass gene-regulatory changes, cell surface remodeling, and immune evasion strategies. Finally, we highlight potential therapeutic avenues such as targeting glycosylation-related pathways, exploiting immunotherapeutic glycopeptide targets, and implementing rational combination regimens. By integrating insights from DTP biology, this review aims to broaden current theories to therapy resistance in lymphoid malignancies and inform the development of innovative treatment strategies.

## 1 Introduction

Lymphoid malignancies are a group of blood tumors derived from lymphocytes, which are characterized by the abnormal proliferation of lymphocytes present in lymph nodes, bone marrow, and various regions, disrupting normal immune function and blood cell production (1, 2). Typical treatment options include chemotherapy, radiation therapy, targeted therapy, and immunotherapy (3, 4). Patients often show a high initial response to chemotherapy but frequently experience relapse due to the persistence of drug-resistant cells.

The cancer stem cell (CSC) hypothesis proposes that a distinct subpopulation of stem-like cells exists within cancers (5–7). In lymphoid malignancies, this concept suggests that disease may arise from cells with self-renewal potential that persist during therapy and contribute to relapse (8). These CSCs have long been regarded as one of the key explanations for therapeutic resistance. Nevertheless, the precise cellular origin of lymphoid malignancies remains under active investigation, and multiple developmental stages or cellular states may contribute to disease initiation and progression (9–12). Thus, while the CSC hypothesis continues to provide valuable insights, additional perspectives are needed to account for the complexity of therapy resistance.

Recent studies highlight drug-tolerant persister (DTP) cells as an emerging complementary model to understand resistance in lymphoid malignancies. DTP cells arise in response to chemotherapy by adopting a quiescent state and remodeling molecular programs and microenvironmental interactions to endure drug exposure (13–15). Unlike genetically fixed resistant clones, DTP cells represent a transient and adaptive residual population, closely linked to minimal residual disease (MRD), that can survive prolonged therapy, escape drug-induced apoptosis, and later regain proliferative capacity after drug withdrawal. Importantly, their resistance mechanisms are largely driven by non-genetic adaptations in recent researches. At the same time, a subset of drug-tolerant expanded persister (DTEP) cells may evolve from DTP cells through subsequent genomic alterations, thereby stabilizing resistance. Insights from extensive research on DTP biology in solid tumors provide a strong foundation for applying these concepts to lymphoid malignancies (16–20). This review summarizes recent advances in the study of DTP cells in lymphoid malignancies. We discuss methods for their induction, detection, and characterization, as well as mechanisms underlying their persistence and potential therapeutic vulnerabilities. In addition, we emphasize the distinctive features of the lymphoid system, including the unique immune interactions. A deeper understanding of DTP is essential for developing more effective therapeutic strategies and eliminating MRD. Especially in lymphoid malignancies, DTP may be an important supplement for CSC hypothesis.

## 2 Origin and research methods of DTP cells in lymphoid malignancies

### 2.1 Drug-tolerant persister cells in cancers

The theory of DTP cells can be traced back to the phenomenon of drug resistance observed during antibiotic therapy. This phenomenon has also been progressively identified in cancer treatment (21) (**Table 1**). Following anticancer drug therapy, some cancer cells can survive under drug pressure, ultimately leading to treatment failure and cancer recurrence. Current research suggests that the emergence of DTP cells primarily occurs through two mechanisms: clonal selection and drug-induced mechanisms (22). The clonal selection mechanism posits that a small fraction of cells with innate drug resistance already exists within the cancer cell population prior to treatment. Due to genomic instability and varying drug sensitivities, these cells may be selected and amplified during drug screening, forming a DTP cell population. This mechanism emphasizes the role of genetic mutations and chromosomal abnormalities in drug tolerance. The drug-induced mechanism posits that DTP cells transform from drug-insensitive cells under therapeutic stimulation. These cells adapt to drug pressure and enter a reversible state of resistance through mechanisms such as epigenetic reprogramming, signaling pathway activation, and altered cellular behavior. This mechanism highlights the critical role of non-genetic factors in DTP cell formation. In recent years, researchers have further proposed multiple mechanisms including epigenetic modifications, signaling pathway remodeling, tumor microenvironment alterations, metabolic reprogramming, and redox regulation (13, 14, 23). These mechanisms collectively confer broad drug tolerance and reversibility on DTP cells. DTP cells have been a significant area of research in cancer therapy (24).

**Table 1:** Summary of various researches related to therapeutic vulnerability of DTP cells.

Type of cancer	Fundamental therapy	Therapeutic vulnerability targets	Therapies targeting DTP cells
A. Directly validated in lymphoid malignancies			
B cell lymphoma (MCL and DHL) (25)	BCL-2 inhibitor (ABT-199)	CDK7, transcriptional machinery	CDK7 inhibitor (THZ1) in combination with ABT-199
Precursor B-cell acute lymphoblastic leukemia (BCP-ALL) (26)	Vincristine, glucocorticoid and asparaginase	ABCC1, CD44, MYH9, NCSTN, KIAA2013, and autophagy/lysosomal function-related genes	ABCC1 inhibitors in combination with vincristine
BCP-ALL (27)	Vincristine, glucocorticoid and asparaginase	Glycocalyx components, (HLA-DRA, CD38, LAMP1, PPT1)	glycosylation enzymes (MGAT3) and specific glycan structures
Childhood BCP-ALL (28)	Chemotherapy (Vincristine and Dexamethasone)	Transcriptional and epigenetic heterogeneity, cell state	Targeting specific cell states (further research is needed)
B. Research on other cancers			
AML (29)	FLT3 inhibitor	Inflammatory signaling	Glucocorticoids
AML (30)	Anthracycline (Doxorubicin)	Histone methylation	KDM6 inhibitior (GSK-J4)
AML (31)	Daunorubicin, Ara-C	Plasma membrane rigidity	Double Negative T cells
Basal cell carcinoma (32)	Hedgehog inhibitor (Vismodegib)	Wnt signaling	Wnt inhibitor (LGK-974)
Breast cancer, prostate cancer (33)	Docetaxel	MYC signaling	CDK9 inhibitor
Breast cancer (34)	Capecitabine	H3K27me3 demethylation	KDM6 inhibitor (GSK-J4)
Breast cancer (35)	BEZ23 and MEK inhibitor (Trametinib)	Bromodomain and extra-terminal domain (BET)	BET inhibitor (JQ1)
Breast cancer (36)	Doxorubicin, cyclophosphamide	Oxidative phosphorylation process	ATP synthase inhibitor (Oligomycin)
Breast cancer, NSCLC, ovarian cancer, melanoma (37)	Tyrosine kinase inhibitor, chemotherapy	Glutathione Peroxidase 4 (GPX4)	GPX4 inhibitor (RSL-3, ML20)
Breast cancer, prostate cancer (38)	Paclitaxel, Adriamycin	NPC1-like transporter 1 (NPC1L1)	NPC1L1 inhibitor (Ezetimibe)
Colorectal cancer (39)	5-Fluorouracil, leucovorin, irinotecan	Autophagic signaling	ULK1 inhibitor (SBI-0206965)
Gastric cancer (17)	5-Fluorouracil	mTOR signaling	mTOR inhibitor (Temsirolimus)
Glioblastoma (40)	PDGFR inhibitor (Dasatinib)	Histone demethylases (KDM6A/B)	KDM6 inhibitor (GSK-J4)
Lung adenocarcinoma (41)	MAPK inhibitor (Trametinib)	Mitophagic signaling	Chloroquine, 3-methyladenine (3-MA)
Lung adenocarcinoma, gastric cancer (42)	EGFR inhibitor (Erlotinib, Crizotinib)	Aldehyde dehydrogenase (ALDH)	ALDH inhibitor (Disulfiram)
Melanoma (43)	Vemurafenib, chemotherapy	Oxidative phosphorylation process	ATP synthase inhibitor (Oligomycin)
Melanoma (44)	Raf inhibitor (Dabrafenib), MEK inhibitor (Trametinib)	RXR signaling	RXR inhibitor (HX531)
Melanoma (45)	BRAF inhibitor (PLX4032), MEK inhibitor (Cobimetinib)	Eukaryotic translation initiation factor 4A1 (eIF4A1)	eIF4A inhibitor (Silvestrol)
Melanoma (46)	Raf inhibitor (PLX4720)	Integrin β1/FAK signaling	FAK inhibitor (PF562271)
NSCLC (47)	EGFR inhibitor (Erlotinib)	H3K9 methylation process	HDAC inhibitor (MS275, TSA)
NSCLC (48)	EGFR inhibitor (Gefitinib)	IGF-1R signaling	IGF-1R inhibitor (AEW541)
NSCLC (49)	Osimertinib, Trametinib	YAP-TEAD signaling	TEAD inhibitor (MYF-01−37)
NSCLC (50)	EGFR inhibitor (Gefitinib, osimertinib)	FGFR3 signaling	FGFR inhibitor (Infigratinib)
NSCLC (51)	EGFR inhibitor (Osimertinib)	AXL signaling	AXL inhibitor (NPS1034)
NSCLC (52)	EGFR inhibitor (Erlotinib)	Wnt signaling	Wnt inhibitor (ICG-001, XAV939)
NSCLC (53)	EGFR inhibitor (Osimertinib, Rosiletinib)	Aurora Kinase A (AURKA)	Aurora inhibitor (MLN8237)
NSCLC (54)	EGFR inhibitor (Erlotinib)	NF-κB signaling	NF-κB inhibitor (PBS-1086)
NSCLC (48)	EGFR inhibitor (Gefitinib)	Histone demethylase 5A (KDM5A)	Trichostatin A (TSA)
NSCLC (55)	EGFR inhibitor (Osimertinib)	BRD4, AURKB, and TEAD	BRD4, AURKB, and TEAD inhibitors
Pancreatic cancer (56)	KRAS ablation	Oxidative phosphorylation process	ATP synthase inhibitor (Oligomycin)

### 2.2 Modeling methods of DTP cells in lymphoid malignancies experiments

The establishment of a model is the beginning of the DTP cells research. Here are some modeling methods DTP cells *in vitro* in experiments of lymphoid malignancies, which have been used in papers (**Table 2**). Researchers co-cultivated human B-cell precursor acute lymphoblastic leukemia (BCP-ALL) cells with mitotically inactivated mouse bone marrow stromal cells (26, 27). To induce drug tolerance, the cells were treated with vincristine at 2 nM and 4 nM concentrations for 18 and 30 days, respectively. The cells were monitored for viability and proliferation during the treatment period. The co-culture system provided a microenvironment that supported the survival and growth of leukemia cells, mimicking the bone marrow environment. In addition, the other team introduced how mantle cell lymphoma (MCL) and double-hit lymphoma (DHL) cells were treated with the BCL-2 inhibitor ABT-199 at concentrations of 20 nM to induce drug tolerance (25). ABT-199 was present while the cells were cultured over several weeks to select DTEP cells. All in all, persisting on drug pressure to screen out the DTP cells is one of the most used methods to induce the DTP model in cancer (29, 30, 57). The method of co-culture is remains an important consideration. Despite these methodological advances, the field of lymphoid malignancy DTP research faces substantial limitations in model availability. To date, well-validated DTP models are restricted to only a narrow subset of lymphoid neoplasms. There is a conspicuous absence of established DTP models for lymphoid malignancies including diffuse large B-cell lymphoma, chronic lymphocytic leukemia, and T-cell malignancies. This constrains the generalizability of current mechanistic conclusions and therapeutic candidate findings.

**Table 2:** A comparison of methods for establishing DTP cell models.

Research	B cell lymphoma (MCL and DHL) (25)	Childhood BCP-ALL (28)	BCP-ALL (27)	BCP-ALL (26)
Establishment Method	Long-term treatment of MCL cell line HBL-2 with ABT-199, screening for DTP cells and expanding into DTEP cells.	Transplantation of childhood BCP-ALL cells into PDX model, followed by chemotherapy with vincristine and dexamethasone.	Co-culture of ICN13 PDX/patient-derived BCP-ALL cells with bone marrow stromal cells, treated with vincristine.	Co-culture of human BCP-ALL cells with bone marrow stromal cells, treated with vincristine for 30 days.
Characteristic	Investigate BCL-2 inhibitor resistance, particularly super-enhancer remodeling and transcriptional reprogramming.	Focus on leukemia cell state heterogeneity, revealing canalization of cell states.	Identify key genes contributing to drug tolerance.	Focus on glycome remodeling of BCP-ALL cells under chemotherapy stress.
Screening and Validation	RNA-seq, ChIP-seq, and drug sensitivity assays.	Single-cell RNA-seq, DNA methylation analysis, and flow cytometry.	RNA-seq and CRISPR screening.	Proteomics, glycomics, and glycoproteomics analyses.

Meanwhile, modeling metastasis *in vivo* is important and the lack of *in vivo* model in DTP cells research is a problem that cannot be ignored (58). Current *in vivo* studies of DTP primarily rely on xenograft models and patient-derived xenograft (PDX) models (59). Compared to static *in vitro* systems, patient-derived xenograft models are better able to capture the dynamic characteristics of persistent states. *In vitro* cultures often lose intratumoral heterogeneity over time and lack physiological tissue architecture. In contrast, patient-derived PDX models preserve the genetic and phenotypic diversity of the primary patient tumor. Recent studies using pediatric BCP-ALL PDX models have shown that chemotherapy induces cellular state differentiation, leading to the emergence of DTP subpopulations with distinct transcriptional profiles—a phenomenon not observed in static *in vitro* single-cell cultures (28). In the future, the further development of *in vivo* experimental models for DTP cell research will enhance our ability to investigate the mechanisms of DTP cell immune evasion and evaluate novel therapeutic strategies targeting these latent subpopulations.

### 2.3 Research methods for DTP cells in lymphoid malignancies

**Figure 1:**
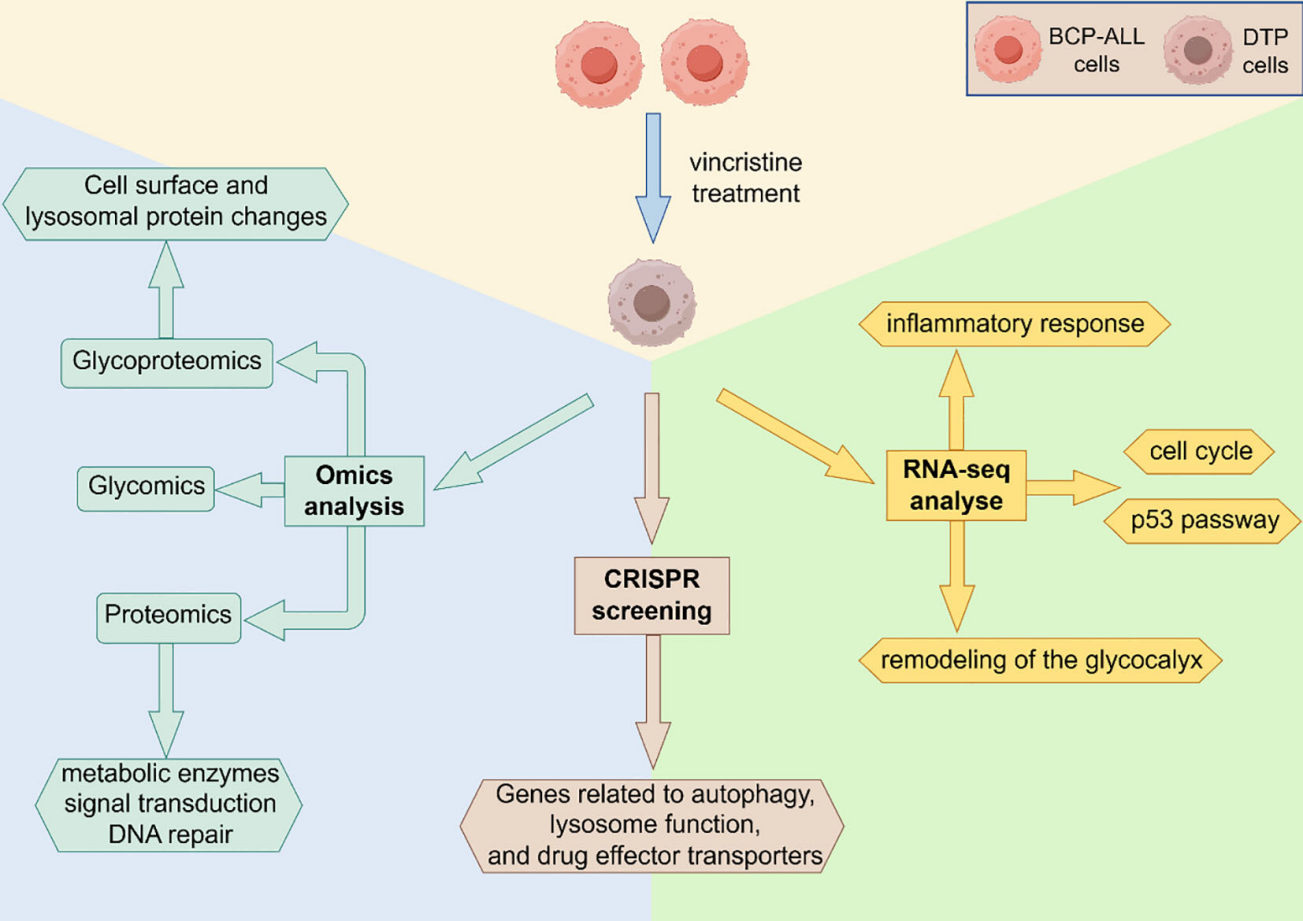
Detection methods of DTP cells in lymphoid malignancies which helpful for carrying out in-depth mechanism researches. Omics analysis, including glycoproteomics, glycomics, and proteomics, identifies alterations in metabolic enzymes, signal transduction, and DNA repair. CRISPR screening reveal genes related to autophagy, lysosome function, and drug effector transporters. RNA-seq analysis elucidates pathways involved in the cell cycle, p53 pathway, remodeling of the glycocalyx, and inflammatory response in lymphoid malignancies. The experimental detection methods and molecular phenotypes shown in the figure have been directly validated in the DTP model of lymphoid malignancies.

Various methods, from the level of molecular and genetic to functional analyses, are used to detect DTP cells (**Figure 1**). RNA-seq allows researchers to comprehensively and quantitatively analyze the RNA transcripts in a biological sample (60, 61). Now, it was used to analyze gene expression changes in DTP cells (37, 62, 63). For example, researchers used it to study gene expression changes in BCP-ALL DTP cells during vincristine treatment (26, 27). Others conducted RNA-seq analysis to explore transcriptome reprogramming in DTEP cells that are resistant to ABT-199 in MCL and DHL (25). In addition, using single-cell RNA-seq, researchers have discovered how MCL evades the immune system and resists drugs (64). Proteomics and glycomics are used to study protein and glycans’ structures, functions, and roles in various biological processes in lymphoid malignancies DTP cells researches (65, 66). For example, researches employed proteomics and glycomics techniques to analyze protein and glycan changes in BCP-ALL DTP cells. Proteomics identified differentially expressed proteins, and glycomics revealed changes in O-glycans, N-glycans, and glycosaminoglycans (27). CRISPR Screening is a high-throughput genetic screening methods that utilize the CRISPR gene-editing technology to systematically perturb genes across the genome and identify those involved in specific biological processes or phenotypes (67–69). Through CRISPR editing, researchers identified genes such as MYH9, NCSTN and KIAA2013 that play roles in survival of BCP-ALL DTP cells (26). The other research used drug sensitivity screens with small-molecule kinase inhibitors and epigenetic drugs to identify vulnerabilities in MCL cells resistant to ABT-199 (27).

## 3 Mechanisms of DTP cells formation and chemoresistance in lymphoid malignancies

Drug resistance mediated by DTP cells arises from a coordinated network of non-genetic adaptations. These mechanisms act synergistically: early transient transcriptional and epigenetic changes may enable cells to survive initially under drug pressure, while stable cell surface remodeling and immune evasion strategies support their long-term survival and ultimately lead to recurrence.

### 3.1 Transcriptomic and epigenetic regulatory changes

#### 3.1.1 Transcriptomic reprogramming of core survival pathways

In cancer drug resistance research, considerable attention has been directed toward mechanisms arising from genetic alterations (70). It may alter the targets of drugs or activate alternative signaling pathways. However, non-genetic mutation resistance mechanisms are crucial, particularly the formation and drug resistant of DTP cells. In BCP-ALL DTP cells, genes related to the p53 pathway, autophagy, and lysosomal function were differentially expressed (27). Together, these changes induce G0/G1 cell cycle arrest and enhance the cells’ ability to recover, enabling them to survive under chemotherapy stress. It indicates that in lymphoid malignancies, the underlying mechanisms of the emergence of DTP cells involve various gene expression changes. In lymphoid malignancies, the B-cell receptor (BCR) signaling pathway frequently experiences dysregulation (71, 72). DTP cells may have altered BCR-associated signaling, which affects their survival and drug resistance. For example, in MCL and DHL, abnormal BCR signaling may interact with the mechanisms of ABT-199 resistance.

#### 3.1.2 Epigenetic regulation of drug tolerance

Histone modifications play a significant role in drug resistance of lymphoid malignancies (73). Research revealed that genes tied to autophagy and lysosomal function aided the survival of BCP-ALL DTP cells, which could be associated with epigenetic regulation such as histone H3K27 trimethylation (27). In addition, the emergence of DTP cells is associated with metabolism-related gene expression changes driven by epigenetic reprogramming.

### 3.2 Adaptive super-enhancer remodeling in DTEP-mediated drug resistance

While transient transcriptomic and epigenetic changes mediate the initial formation of reversible DTP cells, prolonged drug exposure can drive more stable adaptive alterations, including super-enhancer remodeling that promotes the evolution to DTEP cells. BCL-2 is an anti-apoptotic protein crucial in regulating cell survival by inhibiting apoptosis (74, 75). ABT-199 is a BCL-2 inhibitor used in the treatment of lymphoid malignancies (76, 77). Current researchers have found a significant function in MCL from a new angle which focuses on the development of resistance to ABT-199 to understand the mechanisms of drug resistance in MCL and DHL. Although DTP cells are generally considered to lack stable genetic mutations, some studies have reported adaptive genomic alterations, such as loss of the 18q21 amplicon involving the BCL-2 locus, which cooperate with transcriptional reprogramming to mediate resistance (78, 79). The deletion was connected to lower expression of BCL-2 and other apoptotic regulators, including NOXA and TCF4 (80–83). Adaptive super-enhancer remodeling leads to transcriptional reprogramming that drove resistance to ABT-199. In addition, study identified CDK7 as a vulnerability in DTP cells, as inhibition of CDK7 with THZ1 synergized with ABT-199 to overcome resistance (84, 85). Furthermore, functional validation was performed by researchers on significant genes involved in resistance to ABT-199. The overexpression of BCL-2 in DTP cells made them sensitive to ABT-199 again. Conversely, reducing the expression of BCL2A1, MCL-1, and other genes driven by super-enhancers reinstated drug sensitivity (86, 87). These findings highlight the importance of transcriptional and genetic alterations in maintaining drug resistance.

### 3.3 Cell surface proteomic alteration and glycomic remodeling

In addition to intracellular transcriptional reprogramming, DTP cells also undergo extensive remodeling of their cell surface proteome and glycome, which not only modulates intracellular signaling but also mediates interactions with the tumor microenvironment. Protein and glycan play important roles in life activities (88). Proteins are essential macromolecules and glycan is crucial for cellular identification and immune system function (89, 90). The DTP cells in lymphoid malignancies have unique resistance mechanisms because of the cell surface biomarker alterations such as proteomic, glycomic and glycoproteomic remodeling (91, 92). Glycosylation is the predominant modification occurring post-translation on the plasma membrane’s cell surface (93). Based on it, much research has been conducted to investigate the cell surface glycomic and glycoproteomic changes in lymphoid malignancy (94, 95). Research indicates that changes in cell surface proteomic and glycomic structures aid the survival of lymphoid malignancies DTP cells and may be a viable therapeutic target (27). For example, researchers used omics analysis to profile the proteomic and glycomic remodeling of BCP-ALL DTP cells. They suggest that DTP cells have changed in protein expression levels such as lower concentrations of specific metabolic enzymes (26). Significant modifications in the glycome were detected, including a change from Core-2 to Core-1 O-glycans and diminished sialylation (25). Besides, bisecting N-glycans increased, which was associated with higher expression levels of the glycosyltransferase MGAT3 (96). In addition, these changes in glycosylation were associated with altered protein glycosylation, particularly in cell surface and lysosomal proteins such as HLA-DRA, CD38, LAMP1, and PPT1 (97–99). Notably, these cell surface glycomic and proteomic alterations directly contribute to the unique immune evasion strategies employed by lymphoid malignancy DTP cells, as discussed below.

### 3.4 Microenvironment-mediated protection and immune evasion

Lymphoid cells are part of the immune system (100). Based on it, DTP cells in lymphoid malignancies may develop unique mechanisms to evade the immune response. Glycans on the surface of cancer cells might play a role in evading the immune system (101, 102). Changes in the glycosylation of cell surface proteins like HLA-DRA and CD47 may affect immune cell recognition and response (27). For example, altered glycosylation of HLA-DRA impairs antigen presentation to CD4+ T cells, while increased CD47 expression delivers a “don’t eat me” signal to macrophages, enabling DTP cells to evade both adaptive and innate immune surveillance. However, the functional impact of these glycosylation changes on T-cell activation and macrophage phagocytosis has not yet been formally validated in a lymphoid DTP model. The potential mechanistic links have been partially inferred from studies on solid tumors, which indicate that abnormal glycosylation interferes with the recognition of immune cells. In addition, leukemia microenvironment may contribute to immune evasion. Research has shown that co-culture with stromal cells influenced the survival and characteristics of lymphoid malignancies DTP cells in BCP-ALL. However, the exact effects of these mechanisms on DTP cells in lymphoid malignancies require further specialized research although the specific immune regulatory mechanisms mediated by stromal cells have been more thoroughly elucidated in AML and DTP cell models of solid tumors. The microenvironment may also modulate immune surveillance of DTP cells (103). Previous studies have shown that alterations in HLA-DRA and other antigen-presenting molecules may influence immune recognition. However, the immune evasion of DTP cells is likely not limited to changes in HLA expression. DTP cells may interact with T-cell checkpoint pathways such as PD-1/PD-L1 signaling, thereby impairing cytotoxic T-cell activity (104, 105). Natural killer (NK) cell-mediated surveillance is another important but under-explored aspect of DTP biology in lymphoid malignancies. DTP cells may evade the cytotoxic effects of immune killer cells by altering the expression of activation ligands such as NKG2D (106). Although direct evidence linking these checkpoint pathways to DTP cells associated with lymphoid malignancies remains incomplete, they are closely associated with the persistence of MRD. It highlights the need for tailored therapeutic approaches targeting the specific mechanisms of drug resistance in each cancer type.

## 4 From stem cell hypotheses to persister cells in lymphoid malignancies

**Figure 2:**
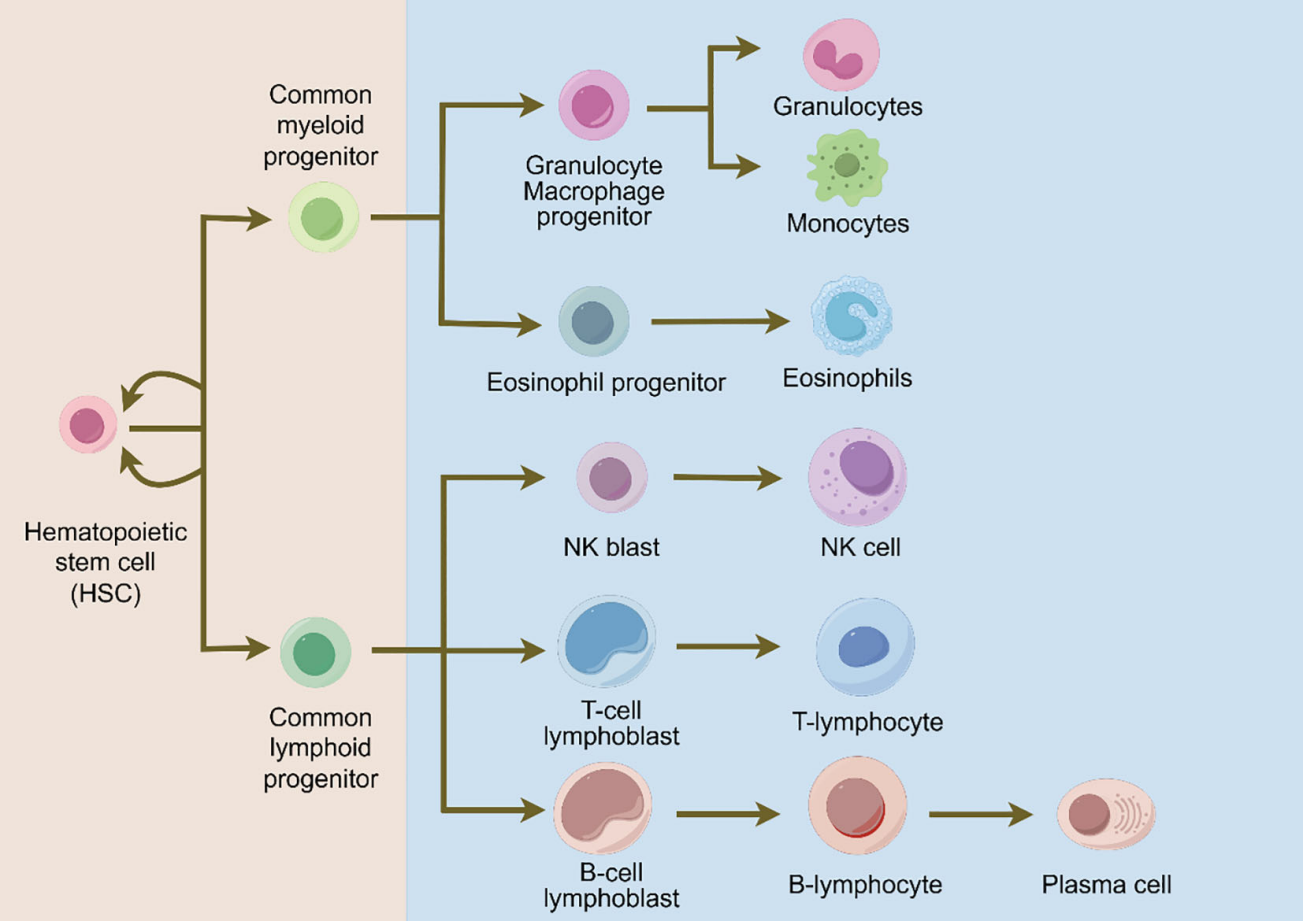
The normal differentiation process of HSC into various blood cell lineages. Internal genes and external signals act together.

Blood cell originates from hematopoietic stem cell (HSC). HSC can generate common myeloid progenitor (CMP) and common lymphoid progenitor (CLP). CMP further develop into granulocytes, monocytes, eosinophils, etc. CLP give rise to NK cell, T cell, B cell, etc. They gradually lose the ability to differentiate into other cell types (107, 108). Now, mature experimental system has been established for the study of blood cell differentiation (109). (**Figure 2**).

**Figure 3:**
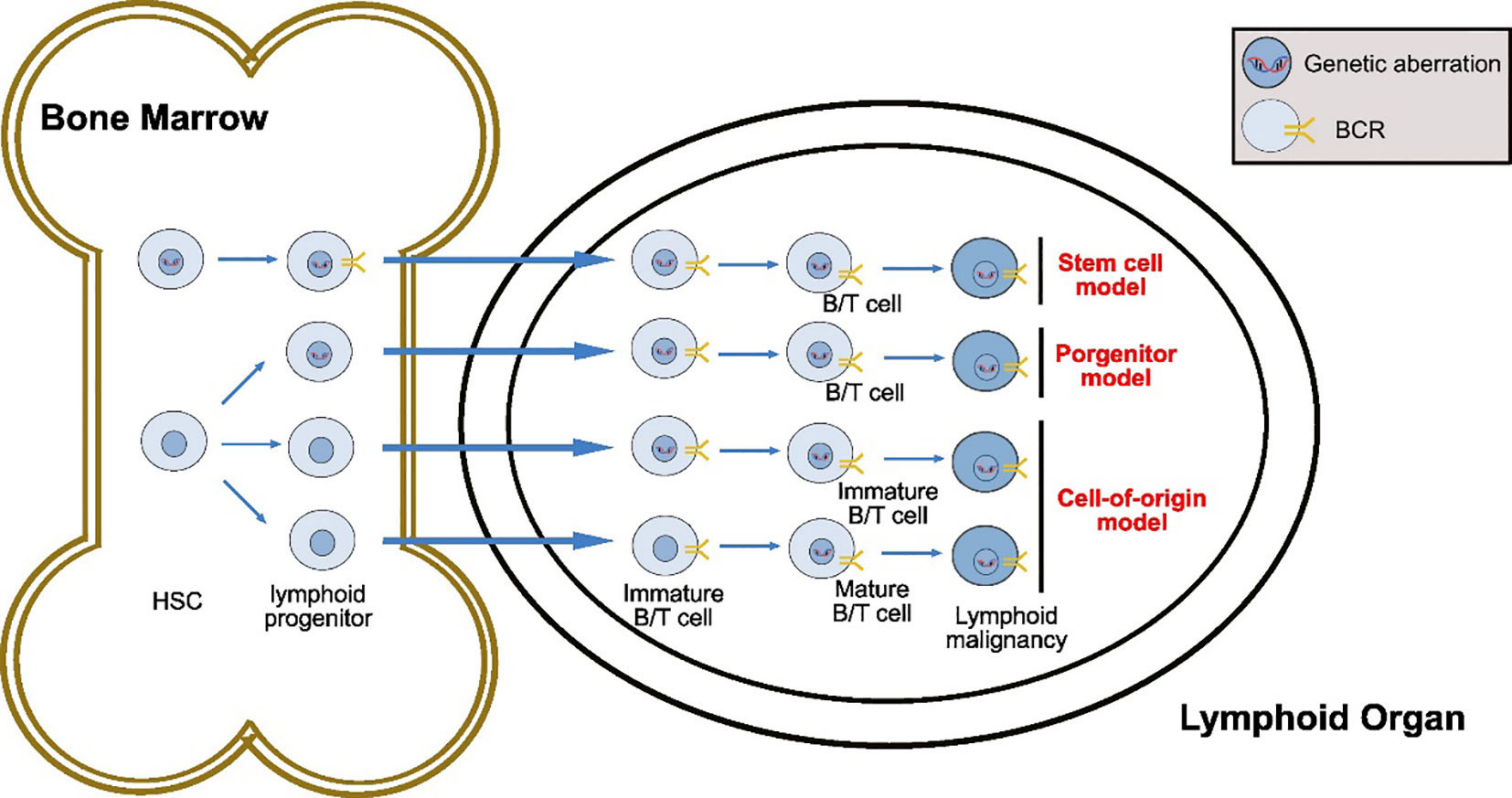
HSC differentiates into lymphoid progenitors in the bone marrow and further develop into immature B/T cells. These cells migrate to lymphoid organs for subsequent maturation progressing through immature and mature phases. There are three hypothesis models about the origin of lymphoid malignancies: the stem cell model suggests genetic aberrations arise in HSC; the progenitor model indicates defects occur in lymphoid progenitors; and the cell-of-origin model proposes that mature B/T cells acquire genetic aberration. These three cell-of-origin models represent established conceptual frameworks for the pathogenesis of lymphoid malignancies but have not yet been directly evaluated in the context of DTP cells.

Base on the CSC hypothesis, the lymphoma stem cell hypothesis suggests that lymphomas originate from stem-like cells that can self-renew and differentiate (110–112). However, this hypothesis remains controversial. On the one hand, according to certain researchers, the proof for lymphoma stem cells is not convincing. The cells identified so far may be subpopulations of tumor cells with enhanced viability. On the other hand, some studies have suggested that lymphomas may occur at different stages of lymphocyte development and that the origin of lymphomas may be more complex (11, 113). In addition, some studies suggest that lymphomas may originate in mature lymphocytes that acquire genetic mutations (9). These mutations transform normal lymphocytes into malignant cells that can self-renew and proliferate. In summary, although the CSC hypothesis provides a paradigm for studying lymphoma recurrence, further studies are needed to clarify exact origin and nature of these cells. (**Figure 3**).

Recent studies of chemoresistance in lymphoid malignancies have revealed new mechanisms such as the emergency of DTP cells. Unlike CSC, DTP cells are not necessarily pre-existing. They can emerge in response to chemotherapy, entering a quiescent state that allows them to survive drug exposure. These cells exhibit plasticity and can dynamically alter their molecular and metabolic profiles to withstand treatment stress. The DTP phenotype is often reversible, meaning these cells can regain proliferative capacity once therapy is discontinued, contributing to disease relapse. DTP cells offer a new angle to explore chemoresistance in lymphoid malignancies, suggesting that therapeutic strategies targeting DTP cells could enhance traditional approaches aimed at CSC.

## 5 Origin of DTP cells and evolution to DTEP cells

DTP cells emerge as a distinct subpopulation of cancer cells capable of enduring chemotherapeutic interventions. Upon chemotherapy exposure, these cells transition into a quiescent state, a cessation of proliferation. In addition, their molecular composition and interactions with the tumor microenvironment have also changed. This adaptive response enables DTP cells to evade drug-induced death and acclimate to the leukemia microenvironment. DTP cells are capable of enduring prolonged chemotherapeutic exposure and, following a period of drug-free recovery, can resume proliferative activity. Collectively, DTP cells represent a dynamic and reversible drug-tolerant subpopulation within cancer cells. The emergence of DTP cells is influenced by factors such as molecular alterations, signaling pathway modifications, and changes in the microenvironment.

**Figure 4:**
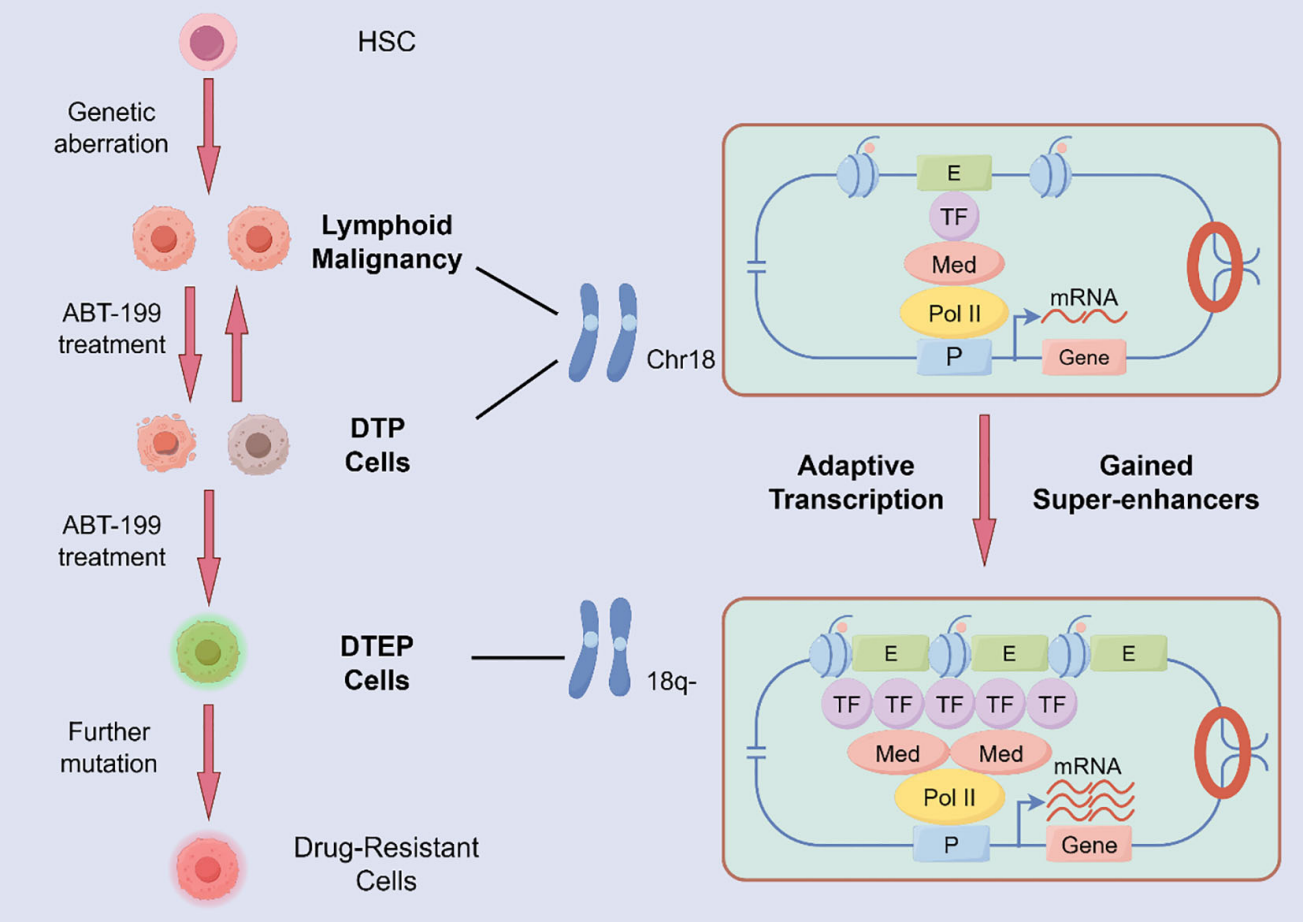
The evolution of drug resistance in lymphoid malignancies through the example of ABT-199 treatment. HSC undergoes multiple complex processes leading to lymphoid malignancies such as the hypothesis models in figure. Upon ABT-199 treatment, DTP cells emerge. Further treatment leads to the evolution from DTP to DTEP cells. These DTEP cells could undergo genomic changes including adaptive transcriptional reprogramming. For example, the gain of super-enhancers drives the transcription of genes associated with drug resistance. The evolutionary trajectory from DTP to DTEP and the deletion of the 18q21 amplicon have been directly confirmed in an ABT-199-resistant MCL/DHL model. The regulatory mechanisms of super-enhancers are supported by both research on lymphoid malignancies and pan-cancer evidence.

DTEP cells act as the subsequent phase in the evolution of DTP cells into more aggressive tumor cells. In our opinion, the concept of DTEP cells harboring genetic mutations does not contradict the canonical definition of DTP cells as having “no stable genomic alterations” (25). DTP cells are defined as a small fraction of tumor cells that enter a quiescent drug-resistant state during drug treatment and can be viewed as minimal residual foci (13, 39). They do not have genomic changes during the initial phase (14). DTEP cells are formed by further evolution DTP cells in the presence of drugs during which cells may undergo genomic changes. For example, the deletion of 18q21 amplicon of DTEP cells led to the downregulation of the apoptosis-regulating gene expression of BCL2, PMAIP1, and TCF4 located on 18q21 and thus made the cells resistant to ABT-199. These specific genomic alterations represent an adaptive response to sustained drug selection pressure. They stabilize the otherwise reversible transcriptional and epigenetic reprogramming in DTP cells, thereby conferring a significant survival advantage to the cells under continuous treatment. Evolutionarily, this is a strategy for tumor cells to evade therapeutic killing and evidenced by the genetic differences between DTP and DTEP cells before and after drug treatment. (**Figure 4**).

The transition to DTEP is not an inevitable outcome for all DTP cell populations. Only a small fraction accumulates stable molecular changes under sustained drug pressure, thereby giving rise to DTEP cells. The interval between the initial formation of DTP and the emergence of stable DTEP or fully resistant cells may represent a “window of opportunity” for clinical therapeutic intervention. It is important to note that the duration of the DTP state likely depends on multiple factors (114). In experimental systems, DTP cells may emerge after prolonged drug exposure ranging from days to weeks, whereas the formation of DTEP cells typically requires more sustained therapeutic pressure and clonal expansion. Therefore, DTP cells may represent an early and potentially reversible stage of residual disease, while DTEP cells may reflect a later stage. During this window of opportunity, interventions targeting the adaptive vulnerability of DTP cells may prevent residual cells from progressing to irreversible resistance. This concept underscores the importance of monitoring MRD rather than waiting for overt relapse (115). Continuous sampling during treatment, after discontinuation, and during the early stages of molecular relapse can help elucidate the process of DTP emergence and progression to DTEP in patients.

## 6 Clinical therapy implications

Various therapeutic strategies targeting DTP cells have been proposed for lymphoid malignancies. In MCL and DHL DTEP models, dual inhibition of CDK7 and BCL-2 has been functionally validated and shown to produce a synergistic antitumor effect. Candidate targets identified through CRISPR screening of BCP-ALL DTP cells have also received direct experimental support in models of lymphoid malignancies including ABCC1, MYH9, and glycosylation-related genes. Other strategies targeting metabolic pathways and immune checkpoints are theoretically promising. However, most of the supporting evidence to date has been derived from other cancer types and requires specific validation in lymphoid DTP models.

Cancer targeted therapy is a treatment method that focuses on cancer cells by disrupting specific molecules that contribute to tumor growth and progression, thus halting cancer’s growth and spread (116–118). New targets which involve metabolism might be a strategy to counteract cancer drug resistance by studies of DTP cells in lymphoid malignancies (119–121). For example, targeting glycosylation-related genes such as MYH9 and NCSTN could enhance the efficacy of chemotherapy (26). The CRISPR screening revealed that genes like C1GALT1C1, SLC33A1, and B3GALNT2, which are involved in glycan synthesis, are essential for the survival of BCP-ALL cells during vincristine treatment (122, 123). In addition, exploring glycopeptides as treatment targets is a potential therapy strategy. For example, chemotherapy-induced intracellular leukemia-specific glycosylation could be a treatment target (124, 125). Recognizing intracellular proteins modified by O-GlcNAc or O-GalNAc as potential neoantigens could pave the way for target and immunotherapy approaches (27, 126). Besides, the gene ABCC1 encodes MRP1, a protein associated with multidrug resistance, which is involved in the efflux of drugs in cancer cells (127, 128). Study suggested that although ABCC1 is not overexpressed in BCP-ALL, it is essential for DTP cells survival during vincristine treatment. Incorporating potential ABCC1 inhibitors during induction therapy may contribute to the elimination of MRD in patients undergoing chemotherapy with vincristine. Genes like MYH9, NCSTN, and KIAA2013 were also identified as important for lymphoid malignancies cell survival (129–131). Targeting these genes could be a potential strategy to eliminate DTP cells especially the function of NCSTN and KIAA2013 are unknown. Furthermore, dual inhibition of CDK7 and BCL2 was found to be an effective strategy (132, 133). THZ1, a CDK7 inhibitor, combined with ABT-199, showed synergistic effects in killing MCL and DHL that are resistant to ABT-199 cells *in vitro* and *in vivo*. This combined treatment can stop the development of resistance to ABT-199 and encourage tumor shrinkage (25).

However, caution must be exercised when considering metabolism-based therapeutic strategies. Normal hematopoietic stem and progenitor cells also rely on tightly regulated metabolic processes (134). Currently, evidence directly demonstrating that DTP cells in malignant tumors possess metabolic characteristics distinctly different from those of normal hematopoietic stem cells remains limited. This issue is particularly important. Therefore, future studies should directly compare DTP cells with normal hematopoietic stem cells to determine whether a potential metabolic vulnerability is specific to DTP cells (135). This would help avoid off-target toxicity during combination therapy.

## 7 Future research direction

Although there are already some detection methods of DTP cells in lymphoid malignancies at present, they need to keep pace with the times (136–138). New detection methods are urgently needed both in experiment and clinical diagnosis as our understanding of lymphoid malignancies deepens. For instance, advanced imaging technologies and high-throughput sequencing methods are being explored to detect DTP cells more precisely. Besides, the study of chromatin accessibility via ATAC-Seq and transcriptional regulation via PRO-Seq is offering new insights into the characteristics and functions of DTP cells in lymphoid malignancies (139, 140).

Future studies should focus on elucidating the molecular mechanisms of DTP cell formation and drug resistant in lymphoid malignancies (141, 142). On the one hand, focusing on the interactions between different signaling pathways in the lymphatic system is important. On the other hand, understanding how DTP cells emerge dynamically is essential. In addition, microenvironmental factors could also potentially influence drug resistance in tumors. Moreover, establishing a new *in vivo* experimental model for DTP cells holds great importance in depth mechanism research of DTP cells in lymphoid malignancies. Current detection methods of DTP cells only be used for experiments. New detection methods are urgently needed to be transformed to clinical application. In addition, the results of preclinical DTP cells studies should be tested in clinical trials at the earliest opportunity to test the efficacy in oncology patients.

In terms of clinical translation, a major challenge is how to standardize the identification of DTP cells in positive MRD patients. The definition of DTP cells is based on a dynamic and reversible state of drug resistance. This makes it difficult to identify them using a single, stable biomarker. Therefore, the clinical identification of DTP cells should integrate evidence from multiple levels. For example, sampling time points should be standardized, including baseline, early treatment, post-treatment MRD assessment, the recovery phase after treatment discontinuation, and the relapse phase. In addition, multiple MRD detection methods should be employed to define residual malignant cell populations. Further refinement is needed in the examination of DTP characteristics in residual cells. More importantly, where feasible, longitudinal analysis should be employed to distinguish DTP states from genetically fixed resistant clones. This would facilitate the potential benefits of early combination therapy before treatment resistance emerges. Future prospective studies are needed to define reproducible DTP characteristics, establish clinically feasible detection methods, and determine whether DTP-targeted therapy can improve the prognosis of lymphoid malignancies.

Cancer treatment needs to be tailored to each individual (110, 143). Recently studies in DTP cells provide new entry angles for personalized patient treatment strategies based on genetic mutations, glycemic profiles and immunological therapy targets. Personalized therapeutic strategies based on the specific mechanisms of lymphoid malignancies may lead to better therapeutic outcomes (2, 144).

## 8 Conclusion

The origins and resistance mechanisms of lymphoid malignancies remain subjects of active research and debate. Both the CSC hypothesis and the DTP cell model offer valuable perspectives, each explaining distinct aspects of disease initiation, persistence, and relapse. Rather than being mutually exclusive, these frameworks may be viewed as complementary, with CSC concepts highlighting the potential role of stem-like populations in disease initiation and long-term maintenance, while DTP biology emphasizes adaptive and reversible states that contribute to therapeutic resistance. Integrating insights from both models may provide a more comprehensive understanding of lymphoid malignancies. In this review, we summarized the emerging roles of DTP cells in lymphoid malignancies, including mechanisms of formation such as glycan remodeling, alterations in gene regulation and signaling pathways, and transcriptional reprogramming. Targeting these processes offers promising avenues for therapeutic intervention, particularly when considered alongside established CSC-related approaches. A combined research strategy that incorporates both CSC and DTP perspectives holds the potential to guide the development of innovative treatments in patients with lymphoid malignancies.
